# An adiabatic method to train binarized artificial neural networks

**DOI:** 10.1038/s41598-021-99191-2

**Published:** 2021-10-05

**Authors:** Yuansheng Zhao, Jiang Xiao

**Affiliations:** 1 Department of Physics and State Key Laboratory of Surface Physics, Fudan University, Shanghai, 200433 China; 2 Department of Physics, University of Tokyo, Tokyo, Japan; 3 Institute for Nanoelectronics Devices and Quantum Computing, Fudan University, Shanghai, 200433 China; 4 Shanghai Qi Zhi Institute, 200232 Shanghai, China

**Keywords:** Computer science, Information technology

## Abstract

An artificial neural network consists of neurons and synapses. Neuron gives output based on its input according to non-linear activation functions such as the Sigmoid, Hyperbolic Tangent (Tanh), or Rectified Linear Unit (ReLU) functions, etc.. Synapses connect the neuron outputs to their inputs with tunable real-valued weights. The most resource-demanding operations in realizing such neural networks are the multiplication and accumulate (MAC) operations that compute the dot product between real-valued outputs from neurons and the synapses weights. The efficiency of neural networks can be drastically enhanced if the neuron outputs and/or the weights can be trained to take binary values $\pm 1$ only, for which the MAC can be replaced by the simple XNOR operations. In this paper, we demonstrate an adiabatic training method that can binarize the fully-connected neural networks and the convolutional neural networks without modifying the network structure and size. This adiabatic training method only requires very minimal changes in training algorithms, and is tested in the following four tasks: the recognition of hand-writing numbers using a usual fully-connected network, the cat-dog recognition and the audio recognition using convolutional neural networks, the image recognition with 10 classes (CIFAR-10) using ResNet-20 and VGG-Small networks. In all tasks, the performance of the binary neural networks trained by the adiabatic method are almost identical to the networks trained using the conventional ReLU or Sigmoid activations with real-valued activations and weights. This adiabatic method can be easily applied to binarize different types of networks, and will increase the computational efficiency considerably and greatly simplify the deployment of neural networks.

## Introduction

Artificial neural networks have dramatically advanced the capability of artificial intelligence in many aspects^1–5^. In artificial neural networks, the most widely used neuron activation functions include the Sigmoid function ${{{\mathrm {sgmd}}\,}}(x) = 1/[1+\exp (-x)]$, the Hyperbolic Tangent function $\tanh (x)$, and the Rectified Linear Unit function ${{{\mathrm {ReLU}}\,}}(x)=\max (x,0)$, etc..^6,7^. All of these activation functions require multiple bits for storage or processing. In contrast, the binary Heaviside step-function, as the simplest nonlinear activation,

1 $$\begin{aligned} {{{\mathrm {H}}\,}}(x)={\left\{ \begin{array}{ll} 1 &{} x\ge 0\\ 0 &{} x<0 \end{array}\right. }, \end{aligned}$$

only takes 1 bit for storing the output. However, the Heaviside function is not suitable for the activation function because of its non-differentiability at $x = 0$ and its vanishing derivatives for $x \ne 0$, which makes it impossible to use the back-propagation algorithm^8^ in the training process. For the same reason, the weights have to be smoothly tunable and cannot take binary values.

Despite of these difficulties, binarization of neural networks is highly desirable. Modern large Deep Neural Networks (DNNs) requires very large memory (hundreds of MB) to store weights and intermediate variables, as well as strong computing power (up to $\sim$ 10 G flops per sample). This is one the of major obstacles limiting the applications of artificial neural networks, especially on mobile devices. However, binarizing weights/activations obviously can provide huge performance boost, thus it is of interest to find ways to achieve binary weights/activations.

Previously, it was believed that the network with binary activation and/or binary weights cannot be trained because good binary weights simply do not exist as the result of inevitable loss of degrees of freedom. However, recently, some pioneering works such as the BinaryConnect^9^ and BNN^10^ proposed by Courbariaux et al. demonstrated that binarization of weights and activation in deep neural network is indeed feasible. During training runs, these methods use binarized weights (deterministically or stochastically) and/or activations (deterministically) for forward pass; for backward pass, Straight-Through Estimator (STE) is used for gradient of activations:

$$\frac{{{\text{d}}\;{\text{sign}}(x)}}{{{\text{d}}x}} = \left\{ \begin{array}{*{20}l}{1} \hfill & {\left| x \right| < 1} \hfill \\ 0 \hfill & {{\text{otherwise }}} \hfill \\ \end{array}\right.$$

and the gradient of binarized weights are applied to raw (un-binarized) weights. These methods can achieve good results of various tasks. Almost all the works on binarization (quantization) relies on this technique, and some improved variants or extensions are proposed, such as XNOR-Nets^11^ that adds a scaling factor to cancel out quantization error, LQ-Nets^12^ that automatically optimizes quantizers, DSQ^13^ which improves STE itself, and DoReFa-Net^14^ which also uses low bit width during backpropagation to reduce training time. Other methods for binarizing (quantizing) weights of neural networks include expectation backpropagation^15^ and Proxquant^16^. Simons and Lee^17^ and Qin et al.^18^ provide reviews of recent progress in binary neural networks.

Apart from binarization or quantization like our work, there are some other efforts to speed up deep neural networks, they use different method from binarization/quantization of networks, but the aim is essentially the same—to boost the performance of neural network. Some examples are using compact layers^19–21^ and compressing network by removing redundant weights^22–25^.

## Method

Here, we describe an efficient yet simple method to train existing networks to take binary activation function and/or binary weights. In this method, we use a parameterized Sigmoid and Hyperbolic Tanh functions with width *w*:

2 $$\begin{aligned} {{{\mathrm {sgmd}}}}_w(x) \equiv [1+\exp (-x/w)]^{-1} \quad {\text{and}}\quad {{\tanh}}_w(x) \equiv {{\tanh}}(x/w) \end{aligned}$$

to approach the Heaviside step-function $\mathrm {H}(x)$ and the sign-function $\mathrm{sign}(x)$ as $w\rightarrow 0$. In order to train the real-valued weights to work with Heaviside-activated neurons, the training process starts with the usual Sigmoid function of finite width $w > 0$, and then *w* is decreased adiabatically until the Sigmoid function becomes a Heaviside-like function. In the end, the network may be trained once more using the Heaviside-function (binary) activations to further stabilize the weights. To achieve binarized weights, the network is slightly modified by replacing the raw weights *W* with the polarized weights: $W\mapsto a \tanh _w(W)$, where *a* is a real-valued constant for each layer, and the polarized weights instead of raw weights are used for the connections between neurons. The raw weights *W* and multiplier *a* are trained as usual. When width *w* is large, the raw weights and polarized weights are identical. But when the width *w* is gradually decreased during the training, the polarized weights become binarized because $a \tanh _{w=0}(W)=\pm a$ as $w\rightarrow 0$. Unlike most of previous works, our method does not require STE to work and may get rid some of its limitations such as gradient mismatch. The parametrized function used in Eq. (2) has some similarity with the Differentiable Soft Quantization (DSQ) method proposed by Gong et al.^13^, but the tunable width used in this work is viewed as a global non-trainable, time-evolving parameter; Proxquant by Bai et al.^16^ used a time-evolving regularizer to binarize weight, but is unable to regularize activation in a similar manner.

For small tasks such as hand written number below, the width *w* can be adjusted manually. For other larger tasks, we adopted one of the following two self-adaptive methods to adjust *w*. *Scheme V1*: a target accuracy is set for each *w*. The network is trained using the current value of *w*. Once the validation accuracy (using the present *w*) reaches target accuracy or the training epochs exceeds 15–30 epochs (exact number depending on tasks), the value of *w* is decreased by a factor of 1.2–2 (depending on tasks, but not crucial) and the learning rate is also reduced by a factor of 1–2. In the mean time, the next target accuracy is set to the present accuracy. *Scheme V2*: there is no target accuracy in this scheme. The width *w* and the learning rate are decreased as in Scheme V1 once the binary validation accuracy (*i.e.*, at $w=0$ instead of at current width) saturates. Note that this method does not require pre-training which is required for V1 and may be viewed as more flexible version of V1.

## Numerical experiments

We use the adiabatic training method described above to binarize the following four different tasks based on some typical neural network structures: (1) a fully-connected network for recognizing hand-written numbers; (2) a convolutional neural network for recognizing the dog-cat pictures; (3) a convolutional neural network for recognizing spoken numbers; (4) a ResNet-20 or VGG-Small neural network for recognizing pictures from CIFAR-10 dataset with 10 classes. We compare the networks obtained from four different supervised training procedures: the conventional *non-binary network* trained with standard Sigmoid or ReLU activations and real-valued weights, the *binary-activation network* trained with Heaviside (binary) activation and real-valued weights, the *binary-weight network* trained with Sigmoid or ReLU activation and binary weights, and the *full-binary network* trained with binary activation and binary weights. All networks are built using the TensorFlow module^26^.

**Figure 1:**
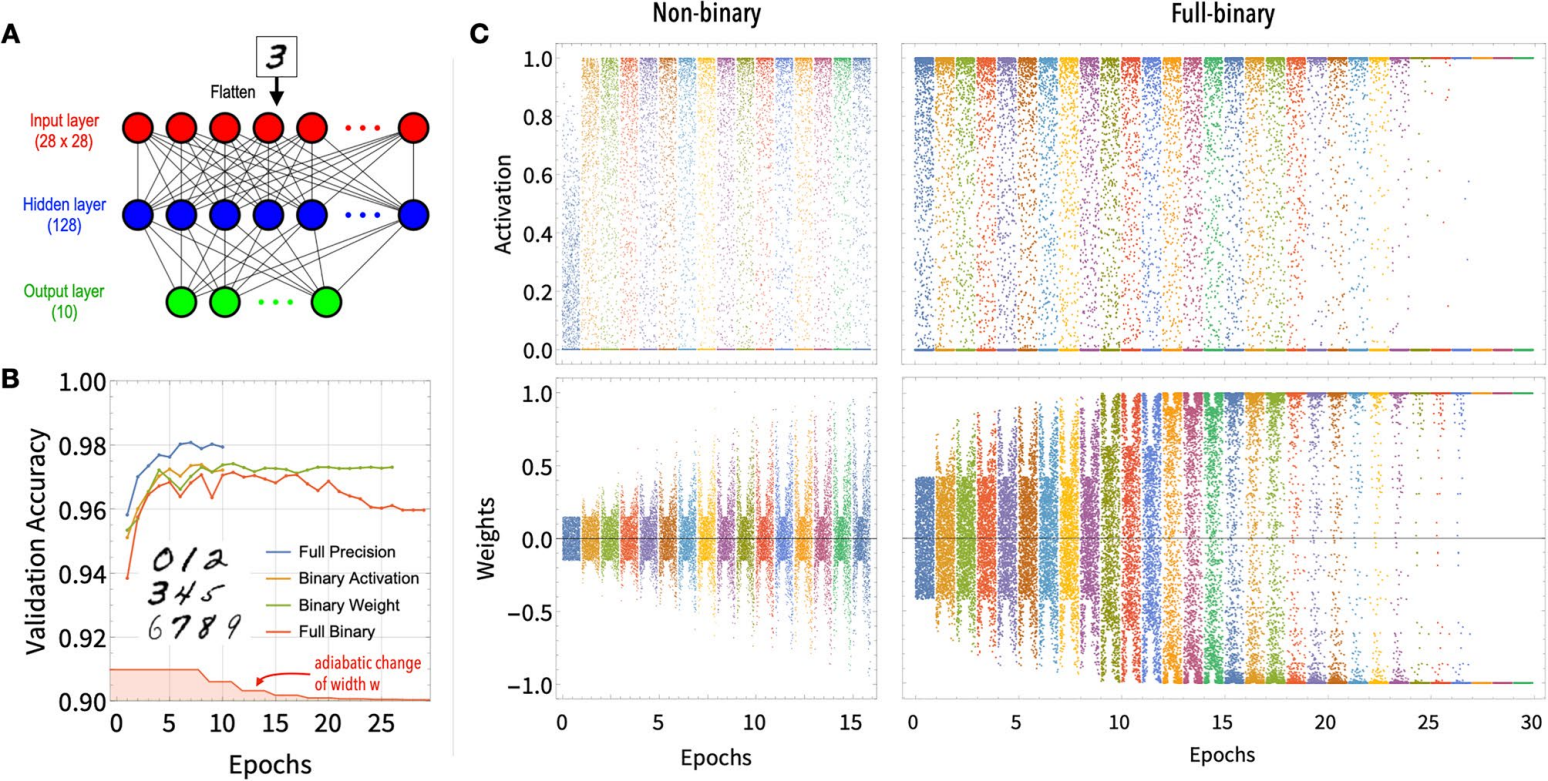
(**A**) The fully-connected neural network with one hidden layer, used for the recognition of hand-written number images. (**B**) The comparison of validating accuracies (using the present width) as function of epochs realized by the *non-binary*, *binary-activation*, *binary-weight*, and *full-binary networks* for the numbered image. The adiabatic change of width *w* as function of epochs for the full-binary case is shown in the bottom of the panel. The sharp dips in the full-binary training curve are due to the sudden change in *w*. (**C**) The distribution of activation outputs (top) and weights values (bottom) for successive epochs (each color band represents one epoch) for the hand-written numbers recognition task (layer 1 only). The left and right panels are results from the non-binary network (with hybrid Sigmoid-ReLU activation function) and the full-binary network, respectively. Each colored band contains 1800 data samples out of $\sim 100\,\mathrm{k}$ weights or 1 million activations. The weights corresponding to the edges of the image remain unchanged during training.

### Image recognition of hand-written numbers

A fully-connected network with one hidden layer (see Fig. 1A) is sufficient for this task^27^. 70 k image samples from the MNIST dataset^28^ are used with 60 k for training/validating and 10 k for testing. drop-out^29,30^ is used to alleviate over-fitting, and no data augmentation is used. For training the non-binary network, the neurons in the hidden layer use the ReLU activation function and 98.2% accuracy can be reached. Since the ReLU function cannot go smoothly to a Heaviside step-function, to train binary networks, we use a hybrid Sigmoid-ReLU activation function as

3 $$\begin{aligned} f_w(x)=2{{{\mathrm {sgmd}}\,}}[{{{\mathrm {ReLU}}\,}}(x)/w]-1, \end{aligned}$$

which can approach the Heaviside function as $w\rightarrow 0$. The width *w* is adjusted manually for this task. The same fully-connected network (of the same size and structure) is trained for 8 epochs using this hybrid activation with $w=1/3$ for neurons in the hidden layer, followed by 3 epochs using $w=1/12$ and 2 epochs with the Heaviside activation. The realized binary-activation network with Heaviside activation has the testing accuracy 97.1% as shown in Fig. 1B, similar to the conventional non-binary network using ReLU activation. It should be noted that the initial epochs of training with finite *w* is crucial, otherwise the validation accuracy can only reach $\sim 85\%$ if using Heaviside activation from start. For the binary-weight network, the network is trained for 8 epochs using the polarized weight with $w=1/3$, followed by 3 epochs each for $w=1/10,1/20,1/50,1/100,1/300,1/500$ and 0, and finally the realized binary-weight network reaches 97.2% testing accuracy as well. The exact sequence of *w* is not important as long as it decreases. For the full-binary network, the network is trained with $w_\text{ weights } = 2 w_\text{ activation }$, and $w_\text{ weights }$ is decreased in the same way as in training the binary-weight network. The final validation accuracy of 96.0% is reached. Therefore, both the half-binary and full-binary networks can reach almost the same validation accuracy as the non-binary network.

Figure 1C shows the distributions of the activation outputs and the weights from the non-binary and full-binary networks. It shows that for the full-binary networks, both the activation outputs and the weights become progressively more and more binarized, and eventually purely binarized at the end of the training process. In comparison, both activations and weights in the non-binary network spread out across the full range for all epochs as expected. In the full-binary case, the weight scaling factor *a* also needs to be trained and is in general a real-valued number, but at the end of the training, *a* can be set to be unity and all activations and/or weights become purely binary. We should mention that it is not desirable to have many weights or activation values close to the center (near zero) of the Sigmoid/Tanh function during training, because the center part cannot be accessed in binarized networks. Therefore, the ratio between the range of the initial weights $\mathrm {Range}(\left| W \right| )$ and the initial value for width *w* should be $\mathrm {Range}(\left| W \right| )/w \gtrsim 1$ (for the first band of Fig. 1C for the full-binary panel, $\mathrm {Range}(\left| W \right| ) \simeq 1/2$ and $w = 1/3$), and in the meantime the learning rate should be moderately large to avoid the center part.

**Figure 2:**
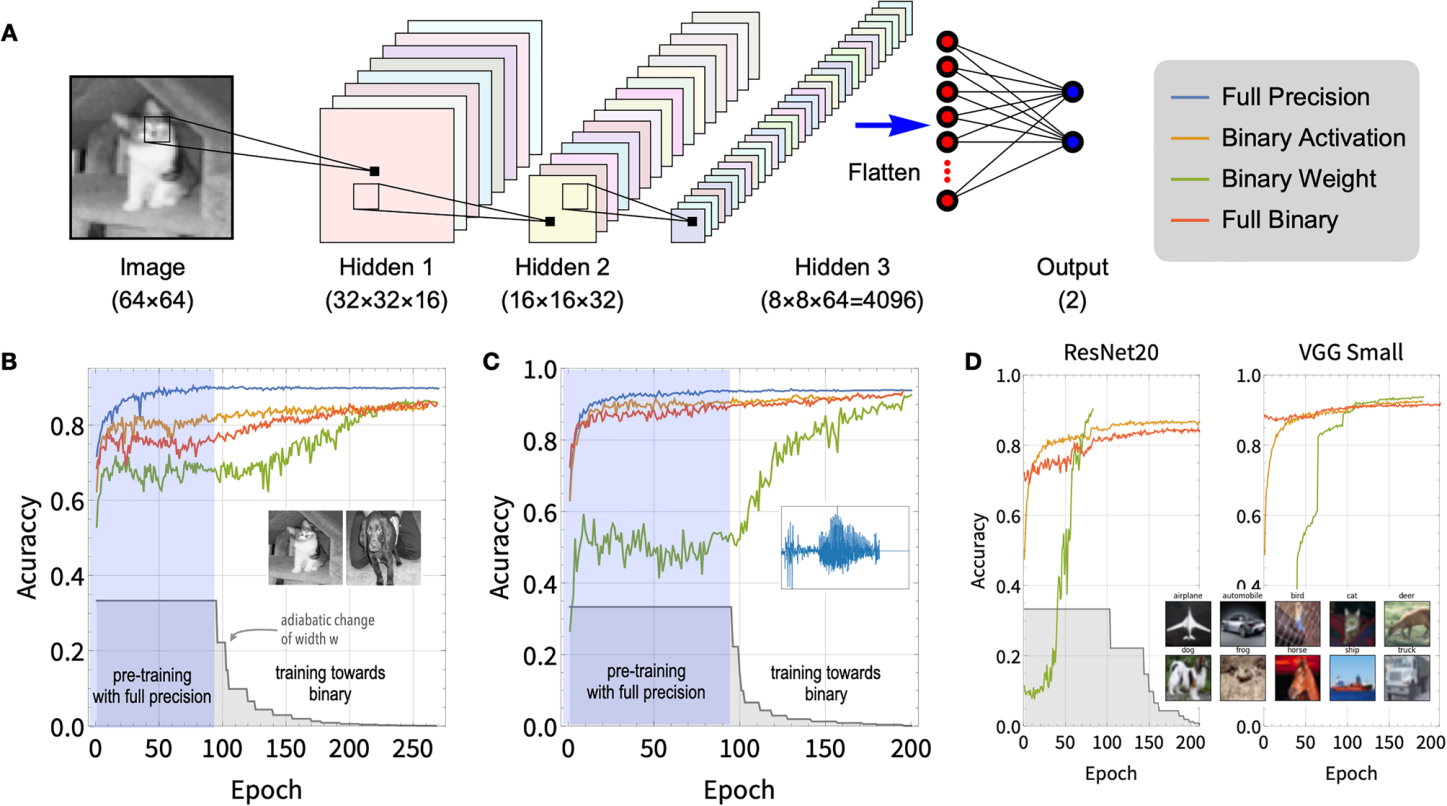
(**A**) The convolutional neural network with three hidden layers used for dog-cat recognition and the spoken number recognition. (**B**) The validating accuracies as function of number of epochs for the dog-cat task using networks trained with usual full precision (blue), binary activation (orange), binary weights (green), and full binary (red). All networks are trained from scratch. The gray stepwise curve shows the adiabatic change of width *w* towards zero as function of epochs. (**C**) Same as (**B**) for the spoken number task. (**D**) results for the CIFAR-10 recognition task based on the ResNet-20 and VGG-Small networks; binary-weight network starts from pre-trained (not shown) model, binary-activation nets are trained from scratch and full-binary nets start from the binary-activation network; for the latter two networks, batch normalization layers work in training mode for evaluation as drop-outs are used (inference mode is used for testing). Binary-weight ResNet-20 stops early because it already reached target accuracy. Full-precision accuracies for these two networks are 92.4% and 93.8%^12^.

### The dog-cat picture recognition

A convolutional neural network (CNN) with three hidden layers (see Fig. 2A) are used for this task. In this network, the convolution kernel is $3\times 3$ and the pooling size is $2\times 2$. And 25 k pictures of dogs and cats from the Kaggle dataset^31^ (subset of Asirra^32^) are used with 23.4 k for training/validating and 2.6 k for testing. The pictures are resized to $64\times 64$ pixels and converted to grayscale before training, the training pictures are randomly flipped horizontally and shifted by a maximum of 8 pixels for data augmentation.

For the conventional non-binary network, the standard ReLU activations with max-pooling are used in all hidden layers, and batch normalization^33^ is used to accelerate training. Within 120 epochs of training, the validation accuracy saturates to 89.7% as shown in Fig. 2B. To train a binary-activation network with the Heaviside activation, the ReLU activation is replaced by the parametrized sigmoid activation Eq. (2) with varying width *w* for the hidden layers except last one which uses the ReLU-sigmoid hybrid activation (using sigmoid here causes mostly 0 output when binarized thus not ideal). Because the max-pooling makes no sense with numbers of 0s and 1s, the order of pooling and activation is changed as convolution $\rightarrow$ batch Normalization $\rightarrow$ pooling $\rightarrow$ activation, the same as^11^. Previously the width is adjusted by hand, but this is not practical for large network. From this task, we use the self-adaptive method V1 mentioned above. With these operations, the realized binary-activation network equipped with Heaviside activation functions reaches a validation accuracy of 84.6% (see the orange curve in Fig. 2B). For a binary-weight network, we use the same method to reduce the width *w* and a validation accuracy of 86.0% is achieved (see the green curve Fig. 2B). In order to realize the full binarization for both activations and weights, we have to leave out the first (weights only) and last layer from being binarized, as in most other methods on binarization like^12,13^. The width *w* for the activations and weights can be reduced to zero alternatively (or simultaneously) to achieve full binarization, and realized a validation accuracy of 85.5% (the red curve in Fig. 2B), close to the non-binary and half-binary networks.

### Voice recognition of spoken numbers

This task uses a similar convolutional neural network (see Fig. 2A) as the one used for the dog-cat recognition task above. The kernel length is 30 and the pool sizes for the three layers are 10, 8, and 5, respectively. And 14 k audio waveforms of human spoken numbers (each resized to array of length 8000) from Speech Commands dataset are used with 13 k for training/validating and 1 k for testing^34,35^, no data augmentation is used for this task. The non-binary network trained using the ReLU activation and real-valued weights along with max-pooling reaches a validation accuracy of 93.7% (see the blue curve in Fig. 2C). Following the same procedure as that used in the dog-cat case, the validating accuracies for the binary-activation (the orange curve in Fig. 2C), binary-weight (the green curve), and full-binary networks (the red curve) trained using the adiabatic method are 91.5%, 91.4%, and 93.0% , all of which are close to the non-binary network. The results on all previous tasks are summarized in Table 1.

### CIFAR-10 classification

In this task, we train a standard ResNet-20^36^ or VGG-Small network^2,12^ (with similar structure as the CNN shown in Fig. 2A) to recognize 60 K (50 K for training/validation and 10 K for testing) $32\times 32$ color images belonging to 10 classes from the CIFAR-10 dataset^37,38^. This task is much more challenging than previous ones. However, the adiabatic method can be applied directly without modification, except that the first and last layer are kept un-binarized for both half- and full-binarization. For the data augmentation, as in^36^, we randomly flip the picture horizontally and shift the image by the maximum of 4 pixels.

Using the adiabatic method and starting from pre-trained full-precision network with ReLU activations, we succeed in training both ResNet-20 and VGG-Small network to function with binary-weight as shown in Fig. 2D, where the validation accuracy of binary-weight network approaches to the accuracy of the full-precision network. The test accuracy reached is 90.2% and 93.3% respectively. Compared with VGG-Small, ResNet-20 is much deeper but with much less feature maps per layer, thus the binarization may cause more information loss. It is known to be very hard to binarize activations. As keeping accuracy while pushing activations to binary value is practically impossible, scheme V2 is used to adjust the width *w*. We successfully train the binary activation and full binary ResNet-20 to testing accuracy 85.7% and 83.0%. A slight modification in network structure by adding shortcut at every conventional layer^39^ can increase the accuracy to 86.2% and 84.1%. On the other hand, VGG-Small networks are more friendly to activation binarization, and binary-activation can be trained using the adiabatic method (adjusting width using method V1) starting with $w=1$ (or pre-training with sigmoid activation) and reached 92.4%. The full-binary VGG-Small networks (see the right panel of Fig. 2D) can also be trained using the same method with accuracy 90.7%. For full-binarization of both ResNet-20 and VGG-Small, starting with weights of binary-activation network can boost accuracy (with $\sim 1\%$ accuracy gain) as binarization of activation is the bottleneck of whole task. Table 1 lists the accuracies for the CIFAR-10 task trained from our method and other existing methods, which shows that the accuracies from our method are better or comparable to the accuracies from other methods, and approach the accuracies from the full-precision network. In the table, methods like BNN and XNOR-Net use relatively vanilla STE for gradient of activation and thus has relatively low accuracy. Improvement on STE like DSQ is more complicated but can increase the accuracy. While CL-BCNN introduces channel wise interaction, effectively changing the network structure, and can achieve highest accuracy. Our method can be viewed as an independent method from STE, simple but still have relatively good performance.

Here, we discuss some tricks and analysis on binarization. Firstly, for parameterized binarizing function for activation, there can be many choice: sigmoid, sigmoid-ReLU hybrid, ${\mathrm {clip}}(x/w,0,1)$, etc.. In early layers, symmetric function usually works better as the mean value and shape of distribution of its output is not largely affected. Secondly, it should be mentioned that the L2 regularization (or weight decay^40^), often used in ResNet-20 or VGG small networks, may reduce the magnitude of raw weights over time or even flip their signs, or may make the network use the center part of sigmoid function which is not accessible after binarized. This may weaken the effect of reducing *w* used in the adiabatic method. From our experiment, when binarizing weights only, this is not problematic at all, but when binarizing activations, the regularizations such as drop-out is recommended in the adiabatic method.

**Figure 3:**
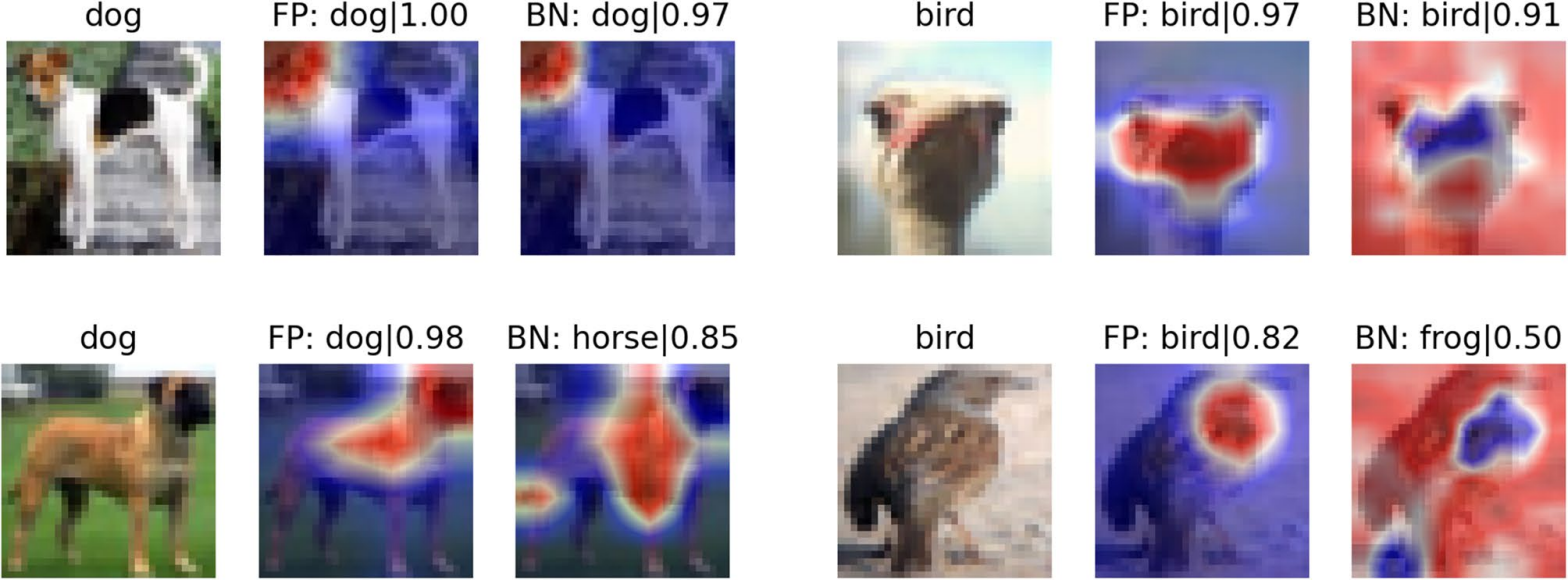
Grad-CAM/CAM of VGG-Small (left) and ResNet-20 (right). In the top row, the images are correctly classified for both full-precision (FP) and full-binary (BN) network, therefore they identify the same defining part of the image. In the bottom row, the images are mis-identified by full-binary network, therefore the BN gives different defining part from the FP network. The numbers are the confidence. Notice that for binary ResNet-20 everything gets reversed.

To see whether the binarized neural network is identifying the same defining part of the image as the full-precission network, we investigated the Gradient-weighted Class Activation Mapping (Grad-CAM)^41^ (for VGG-Small) and usual CAM (for ResNet-20) of full-precision and full-binary network, as shown in Fig. 3. We find that the neural network indeed focuses on the defining part of the image that it correctly identifies. However, for full-binary ResNet-20, we find that the all the weights in the last layer (fully connective layer) are negative, and the heat map is reversed. This suggests that the structure of weights in binary ResNet-20 may be completely different from its full-precision counterpart and can be the reason that ResNet-20 is very difficult to binarize activations.

**Table 1:** Comparison of accuracy for all tasks.

Tasks	Networks	Method	Binarization	*w*-Reduction	Accuracy (%)
MNIST	2-layer DNN		Full precision		98.2 (0.1)
		Ours	Weights only	Manual	97.2 (0.1)
		Ours	Activation only	Manual	97.1 (0.1)
		Ours	Weights and activations	Manual	96.0 (0.2)
Dogs vs Cats	4-layer CNN		Full precision		89.7 (0.2)
		Ours	Weights only	V1	86.0 (0.3)
		Ours	Activation only	V1	84.6 (0.1)
		Ours	Weights and activations	V1	85.5 (0.6)
Spoken numbers	4-layer CNN		Full precision		93.7 (0.2)
		Ours	Weights only	V1	91.5 (0.5)
		Ours	Activation only	V1	91.4 (0.8)
		Ours	Weights and activations	V1	93.0 (0.2)
CIFAR-10	ResNet-20		Full precision		92.4^12^
		Ours	Weights only	V1	90.2 (0.1)
		DoReFa^14^	Weights only		90.0
		LQ-Net^12^	Weights only		90.1
		DSQ^13^	Weights only		90.2
		ProxQuant^16^	Weights only		90.7
		Ours	Activation only	V2	86.2 (0.3)
		Ours	Weights and activations	V2	84.1 (0.2)
		DoReFa^14^	Weights and activations		79.9
		DSQ^13^	Weights and activations		84.1
		CL-BCNN^42^	Weights and activations		91.1
	VGG-Small		Full precision		93.8^12^
		Ours	Weights only	V1	93.3 (0.1)
		BWN^11^	Weights only		90.1
		BinaryConnect^9^	Weights only		91.7
		LQ-Net^12^	Weights only		93.5
		Ours	Activation only	V1	92.4 (0.2)
		Ours	Weights and activations	V1	90.7 (0.2)
		XNOR-Net^11^	Weights and activations		89.8
		BNN^10^	Weights and activations		89.9
		DSQ^13^	Weights and activations		91.7
		CL-BCNN^42^	Weights and activations		92.5

The classes in all datasets are completely balanced.

## Discussion and conclusion

With the four different tasks demonstrated above, we showed that, by adiabatically varying the width of the activation and weight distribution towards zero, the existing neural networks can be trained to work with binarized Heaviside activated neurons and/or binarized weights. And the realized binary networks have the validation accuracy approaching that obtained from the conventional non-binary neural networks. The most distinguishing advantage of this adiabatic approach for training binary networks is its applicability to many (if not all) existing neural networks with no or slight change in terms of network size or structure. Reflecting in the coding, this corresponds to a few lines of code change in the training programs (see the program codes^43^). Our method can also be applied in conjunction with other existing tricks (e.g. adding channel wise interaction like^42^) to obtain better results.

Compared with the non-binary neural networks, the benefits of binary network are obvious. Firstly, a Heaviside activation simplifies the neuron to a 1-bit yes or no decision maker, which would greatly speed-up and simplify both software- and hardware-implemented neuron realizations. For example, a CMOS based hardware-implemented neuron takes several dozens of transistors to construct a sigmoid-activated neuron, but it only requires one transistor for a Heaviside-activated neuron. Binarized weight can heavily reduce the storage along with computation burdens. Compared with a float32 type weight, binary weights only takes about 1/32 of storage space (Although this needs specialized hardware design). For VGG-Small, there are 4.67 M parameters, costing 19 MB memory storage. Binarization, can eliminate 4.57 M full precision weights, thus reduces storage to less than 1 MB. The memory saving on larger neural network will be more drastic. Secondly but more dramatically, the most time/area/energy-consuming part of a conventional non-binary neural network is the weight readout and the multiply-accumulate (MAC) operation^44^ which calculates the dot product of the outputs of previous layer neurons and the weights. If the number of neurons involved is *N*, then one MAC operation includes *N* multiplication and *N* addition operations. In VGG-Small, $1.2\times 10^9$ flops are required for each sample. However, for a binary-activation neural network, since the neuron outputs are either 0 or 1, the matrix multiplication operation is completely unnecessary, one needs only sum over those weights associated with neuron output 1 and omitting all the weights corresponding to zero outputs. The binarized weights in a binary-weight network can reduce matrix multiplication for a similar reason. For full-binary neural networks, since both neuron outputs and the weights are binary, the addition operation is further simplified to a single type of counting (XNOR) operation (Strictly speaking, only multiplication between $\pm 1$ is equal to XNOR, however, multiplication between $\pm 1$ weights and any binary activation only differs by a bias term, which can be absorbed into batch normalization layer.). Full binarized VGG-Small network only requires $3\times 10^5$ flops per sample for un-binarized first and last layers as well as batch normalization. The speed up is tested to be around 7 times faster according to other works^10,39^. Because the Heaviside activation function simply turns on or off the contributions from some of the weights, there is no need to read out the weights corresponding to the off-neuron, which reduces the read-out operations from *N* to $\sim N/2$ on average. Because of these obvious benefits, the binarized neural network can have huge advantage in the deployment.

## References

[1] Krizhevsky A, Sutskever I, Hinton GE (2012). Imagenet classification with deep convolutional neural networks. Adv. Neural. Inf. Process. Syst..

[2] Simonyan, K., & Zisserman, A. Very deep convolutional networks for large-scale image recognition. arXiv:1409.1556 (arXiv preprint) (2014).

[3] Hinton G, Deng L, Yu D, Dahl GE, Mohamed A-R, Jaitly N, Senior A, Vanhoucke V, Nguyen P, Sainath TN (2012). Deep neural networks for acoustic modeling in speech recognition: The shared views of four research groups. IEEE Signal Process. Mag..

[4] Bahdanau, D., Cho, K. & Bengio, Y. Neural machine translation by jointly learning to align and translate. arXiv:1409.0473 (arXiv preprint) (2014).

[5] Silver D, Huang A, Maddison CJ, Guez A, Sifre L, Van Den Driessche G, Schrittwieser J, Antonoglou I, Panneershelvam V, Lanctot M (2016). Mastering the game of go with deep neural networks and tree search. Nature.

[6] Kröse B, van der Smagt P (1993). An Introduction to Neural Networks.

[7] Goodfellow I, Bengio Y, Courville A, Bengio Y (2016). Deep Learning.

[8] Rumelhart DE, Hinton GE, Williams RJ (1986). Learning representations by back-propagating errors. Nature.

[9] Courbariaux M, Bengio Y, David J-P (2015). Binaryconnect: Training deep neural networks with binary weights during propagations. Adv. Neural Inf. Process. Syst..

[10] Hubara I, Courbariaux M, Soudry D, El-Yaniv R, Bengio Y (2016). Binarized neural networks. Adv. Neural Inf. Process. Syst..

[11] Rastegari, M., Ordonez, V., Redmon, J. & Farhadi, A. Xnor-net: Imagenet classification using binary convolutional neural networks. In *European Conference on Computer Vision*, 525–542 (Springer, 2016).

[12] Zhang, D., Yang, J., Ye, D. & Hua, G. Lq-nets: Learned quantization for highly accurate and compact deep neural networks. In *Proceedings of the European Conference on Computer Vision (ECCV)*, pp. 365–382 (2018).

[13] Gong, R., *et al.* Differentiable soft quantization: Bridging full-precision and low-bit neural networks. In *Proceedings of the IEEE International Conference on Computer Vision*, pp. 4852–4861 (2019).

[14] Zhou, S., Wu, Y., Ni, Z., Zhou, X., Wen, H. & Zou, Y. Dorefa-net: Training low bitwidth convolutional neural networks with low bitwidth gradients. arXiv:1606.06160 (arXiv preprint) (2016).

[15] Soudry D, Hubara I, Meir R (2014). Expectation backpropagation: Parameter-free training of multilayer neural networks with continuous or discrete weights. Adv. Neural Inf. Process. Syst..

[16] Bai, Y., Wang, Y.-X. & Liberty, E. Proxquant: Quantized neural networks via proximal operators. arXiv:1810.00861 (arXiv preprint) (2018).

[17] Simons T, Lee D-J (2019). A review of binarized neural networks. Electronics.

[18] Qin H, Gong R, Liu X, Bai X, Song J, Sebe N (2020). Binary neural networks: A survey. Pattern Recogn..

[19] Howard, A. G., *et al.* Mobilenets: Efficient convolutional neural networks for mobile vision applications. CoRR, arXiv:abs/1704.04861 (2017).

[20] Iandola, F. N., *et al.* Squeezenet: Alexnet-level accuracy with 50x fewer parameters and $$<1\text{mb}$$ model size. CoRR, arXiv:abs/1602.07360 (2016).

[21] Szegedy, C., *et al.* Going deeper with convolutions. CoRR, arXiv:abs/1409.4842 (2014).

[22] Denil M, Shakibi B, Dinh L, Ranzato MA, de Freitas N, Burges CJC, Bottou L, Welling M, Ghahramani Z, Weinberger KQ (2013). Predicting parameters in deep learning. Advances in Neural Information Processing Systems.

[23] Han, S., Pool, J., Tran, J. & Dally, W. J. Learning both weights and connections for efficient neural networks. arXiv:1506.02626 (arXiv preprint) (2015).

[24] He, Y., Zhang, X. & Sun, J. Channel pruning for accelerating very deep neural networks. In *Proceedings of the IEEE International Conference on Computer Vision*, pp. 1389–1397 (2017).

[25] Liu, B., Wang, M., Foroosh, H., Tappen, M. & Pensky, M. Sparse convolutional neural networks. In *Proceedings of the IEEE Conference on Computer Vision and Pattern Recognition*, pp. 806–814 (2015).

[26] TensorFlow: https://www.tensorflow.org/.

[27] LeCun Y, Bottou L, Bengio Y, Haffner P (1998). Gradient-based learning applied to document recognition. Proc. IEEE.

[28] http://yann.lecun.com/exdb/mnist/.

[29] Srivastava N (2013). Improving neural networks with dropout. Univ. Toronto.

[30] Srivastava N, Hinton G, Krizhevsky A, Sutskever I, Salakhutdinov R (2014). Dropout: A simple way to prevent neural networks from overfitting. J. Mach. Learn. Res..

[31] https://www.kaggle.com/c/dogs-vs-cats/data.

[32] Elson, J., Douceur, J. J., Howell, J.& Saul, J. Asirra: A captcha that exploits interest-aligned manual image categorization. In *Proceedings of 14th ACM Conference on Computer and Communications Security (CCS), Association for Computing Machinery, Inc.* (2007).

[33] Ioffe, S. & Szegedy, C. Batch normalization: Accelerating deep network training by reducing internal covariate shift. arXiv:1502.03167 (arXiv preprint) (2015).

[34] Warden, P. Speech commands: A dataset for limited-vocabulary speech recognition. arXiv e-prints (2018).

[35] https://www.kaggle.com/mok0na/speech-commands-v002.

[36] He, K., Zhang, X., Ren, S.& Sun, J. Deep residual learning for image recognition. In *Proceedings of the IEEE Conference on Computer Vision and Pattern Recognition*, pp. 770–778 (2016).

[37] https://www.cs.toronto.edu/~kriz/cifar.html.

[38] Krizhevsky, A. & Hinton, G. Learning Multiple Layers of Features from Tiny Images. Technical Report, University of Toronto, Toronto (2009).

[39] Liu, Z., *et al.* Bi-real net: Enhancing the performance of 1-bit cnns with improved representational capability and advanced training algorithm. In *Proceedings of the European Conference on Computer Vision (ECCV)*, pp. 722–737 (2018).

[40] Hanson, S. J. & Pratt, L. Y. Comparing biases for minimal network construction with back-propagation. In *Proceedings of the 1st International Conference on Neural Information Processing Systems*, pp. 177–185 (1988).

[41] Selvaraju, R. R., Cogswell, M., Das, A., Vedantam, R., Parikh, D. & Batra, D Grad-cam: Visual explanations from deep networks via gradient-based localization. In *Proceedings of the IEEE International Conference on Computer Vision*, pp. 618–626 (2017).

[42] Wang, Z., Lu, J., Tao, C., Zhou, J. & Tian, Q. Learning channel-wise interactions for binary convolutional neural networks. In *Proceedings of the IEEE/CVF Conference on Computer Vision and Pattern Recognition*, pp. 568–577 (2019).

[43] https://github.com/YuanshengZhao/adiabaticbinary.

[44] Sze V, Chen Y-H, Yang T-J, Emer JS (2017). Efficient processing of deep neural networks: A tutorial and survey. Proc. IEEE.

